# A generalised deep learning-based surrogate model for homogenisation utilising material property encoding and physics-based bounds

**DOI:** 10.1038/s41598-023-34823-3

**Published:** 2023-06-05

**Authors:** Rajesh Nakka, Dineshkumar Harursampath, Sathiskumar A Ponnusami

**Affiliations:** 1 NMCAD Laboratory, Department of Aerospace Engineering, Indian Institute of Science, Bengaluru, Karnataka India; 2 Aeronautics and Aerospace Research Centre, Department of Engineering, City, University of London, Northampton Square, London, UK

**Keywords:** Composites, Mechanical properties, Aerospace engineering, Mechanical engineering, Computational methods

## Abstract

The use of surrogate models based on Convolutional Neural Networks (CNN) is increasing significantly in microstructure analysis and property predictions. One of the shortcomings of the existing models is their limitation in feeding the material information. In this context, a simple method is developed for encoding material properties into the microstructure image so that the model learns material information in addition to the structure-property relationship. These ideas are demonstrated by developing a CNN model that can be used for fibre-reinforced composite materials with a ratio of elastic moduli of the fibre to the matrix between 5 and 250 and fibre volume fractions between 25 and 75%, which span end-to-end practical range. The learning convergence curves, with mean absolute percentage error as the metric of interest, are used to find the optimal number of training samples and demonstrate the model performance. The generality of the trained model is showcased through its predictions on completely unseen microstructures whose samples are drawn from the extrapolated domain of the fibre volume fractions and elastic moduli contrasts. Also, in order to make the predictions physically admissible, models are trained by enforcing Hashin–Shtrikman bounds which led to enhanced model performance in the extrapolated domain.

## Introduction

Machine learning (ML) models, especially its subdomain artificial neural networks (ANN), were proved to be valuable tools in the design and analysis of composite materials^1–3^. First, these models are developed by learning from the data points generated by simulations or collected from experiments. Later, during deployment, this model is used to make inferences about any data point with the same characteristics as those used during learning. Generally, the initial model development process involves computational costs (memory and time) for generating data and training the model. The expected advantage is that, with the developed model, predictions can be made in significantly shorter times. Here, the number of points needed for training a model depends on multiple factors such as the amount of prior knowledge of the system used in the training process^4^, the complexity of input-output relation and the expected accuracy of the model. Active research is focused on utilising known physics, like governing or constitutive equations, during model training. In this direction, physics-informed neural networks (PINNs)^5–7^ have gained much attention for accurately solving the PDEs of the underlying physics. A knowledge-based sampling of inputs is another way of utilising the physics of the problem in model training^8,9^. In addition to prior knowledge infusion, the type of ANN architecture plays an essential role in effective and effortless learning. Some of the successful ANN architectures include; convolutional neural networks (CNN) for image kind of data, recurrent neural networks (RNN) for sequential or time-series data and generative adversarial networks (GAN) for learning the distribution of the given data.

Evaluation of composite material properties is a non-trivial task due to heterogeneities at various length scales and the statistical nature of the constituent’s distribution and morphology. As the experimental methods are time-consuming and economically costlier, analytical solutions are developed to find the properties of an equivalent hypothetical homogeneous material that responds similarly to the composite material. These solutions are obtained by certain assumptions, thus only applicable to simpler cases with restrictions on constituent geometry and distribution. These shortcomings can be addressed with finite element analysis (FEA) based homogenisation^10,11^, where multiple boundary value problems are solved on a representative volume element (RVE) using different load cases. Some variations of this conventional FEA approach^12,13^ are developed to reduce the computational costs. Variational Asymptotic Method (VAM)-based homogenisation, for example, gives an effective material matrix using single finite element analysis without any post-processing in contrast to solving multiple cases along with equally demanding post-processing steps in the conventional approach. Still, the computational time and resources required are significant enough to slow down the search for better composite materials. Hence, active research is being pursued to combine computational micro-mechanics and data-driven artificial intelligence (AI) methods to build surrogate models^6,7,14–17^.

CNN models have been used widely in the micro-mechanics^15,16,18–21^ as micro-structure information is available, generally, either in image form (for 2D) or voxelated form (for 3D). The success of CNN architecture over simple artificial neural networks can be attributed to its self-feature learning capability and utilising local connectivity characteristics using the following two basic assumptions^22^. One, low-level features are assumed to be local and do not depend on the spatially far-off features, which is implemented by connecting downstream neurons with only spatially neighbouring neurons in the upstream through a kernel (or filter) of the convolution operation. The second assumption is that a feature learnt at one spatial location is useful at the other location. Hence the kernel with the same weights is used at all locations of the image. Generally, CNN models are constructed in two stages. First, data features are learnt through a series of convolution and pooling operations on the input samples. The second stage contains a conventional multi-layer perceptron which takes the output of the first stage as a flattened array. Dense connections of the last stage increase the number of learnable parameters drastically, thus leading to heavier computation costs and longer training times. Hence, Mann and Kaidindi^20^ have developed a CNN model wherein the output of the first stage is directly mapped to outputs. Also, at the end of the first stage, using globally averaged pooling instead of simple flattening was proved to reduce the number of parameters and over-fitting in the model^18,23^. Innovative architectures of the first stage have led to efficient CNN models like AlexNet, VGG, and ResNet. Among these, the VGG model has been adopted widely in many micro-mechanical models^18,19,24^, either directly by transfer learning or using its principle of stacking convolutional layers with delayed pooling operations. For example, Li et al.^19^ used pruned VGG-16 model for learning and reconstructing micro-structure features wherein high-level layers or those away from the input layer are removed to reduce the computational cost. We have used the working principle of this simple and standard architecture because the primary focus of the present work is to develop data sets that are aware of the material information and to evaluate its influence on the model performance. Though CNN models are free of feature engineering, some models have demonstrated that by supplying modified input instead of simple raw images, model learning capability can be enhanced^17,20,25^. For example, Mann and Kalidindi^20^ used two-point spatial correlations of the micro-structure; Cheng and Wagner^17^ have developed RVE-net which uses loading conditions and parameterised geometry (by level set fields) as the input. As the preparation of labels is computationally intensive, some CNN models have developed using physics information to learn labels implicitly^17,26^. Li and Chen^26^ have modelled the constitutive behaviour of the hyper-elastic materials by embedding equilibrium conditions in the CNN model.

In the case of composite materials, it is desirable to have a surrogate model that can be used across more comprehensive ranges of fibre volume fractions ($V_f$) and constituent properties. The existing models are built for either a particular fibre volume fraction or a small range of fibre volume fractions (less than 50%) and a particular fibre-matrix materials combination. In this work, we develop a model that can be used for broader ranges of fibre volume fractions $V_f \in [25\%, 75\%]$ and fibre-matrix elastic modulus contrast (ratio) $E_{cr} \in [5, 250]$ and also the predictive capabilities of the trained models is assessed in the extrapolated domain of $V_f \in [10\%, 75\%]$ and $E_{cr} \in [5, 500]$. Grayscale images of the microstructure provide the geometrical features like $V_f$ but not the material information. So, if the model has to work with different material systems, it should learn to detect constituent material properties. For this purpose, a simple and novel method is developed wherein material information is supplied as higher-order tensors which are prepared by encoding material properties of each phase into a grayscale image of the microstructure. Another alternative way of ingesting the constituent properties is through multi-modal or mixed inputs. In this approach, numerical values of the constituent properties can be concatenated to the flattened array after the convolution operation, avoiding the encoding operation^27^. However, this approach might require more samples to learn the spatial location of the material properties, whereas the samples prepared from direct encoding are informed about the constituent’s spatial location. Also, the physical admissibility of the model predictions is assessed using physics-based bounds. Despite the acceptable levels of performance metrics, a significant number of outliers to bounds are observed in certain regions of the domain. These outliers are completely eliminated by training the models with hard enforcement of bounds. For this purpose, we have used the Hashin–Shtrikman bounds^28,29^ in model training.

The paper is structured as follows: initially, datasets generation is explained with the details of microstructure generation, material property encoding and label preparation. Then, CNN models are built, and their performance is studied on the unseen samples of the training data sets domain and their extrapolated domain using absolute percentage error plots. In the end, the physics-based bounds are used to quantify and eliminate the physically inadmissible model predictions.

## Data sets generation

**Figure 1:**
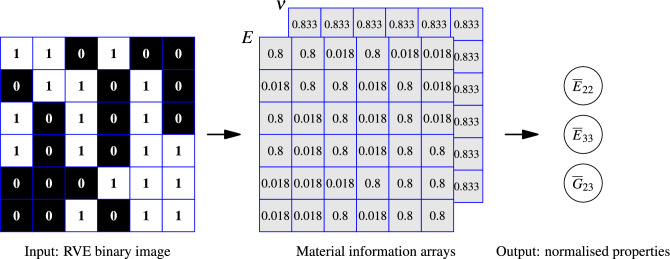
Schematic of data set elements showing RVE binary image (input to the model), material information arrays (prepared at the beginning of the model inference) and transverse elastic properties normalised with the respective matrix modulus (output of the model).

The dataset is constituted of a stack of RVE samples wherein each sample contains the binary image of the RVE as input and its normalised transverse elastic properties as target labels. Here, RVE is a representative volume element of the unidirectional composite with randomly distributed fibres of circular cross-sections. Let ${\mathscr {X}}_{bw} \in {\mathbb {R}}^{n_s \times n_w \times n_h \times 1}$ be the input part of the dataset containing $n_s$ number of RVE images with $n_w$ and $n_h$ pixels along width and height, respectively. Along with ${\mathscr {X}}_{bw}$, one needs to supply material properties of the constituents, which will be encoded into the RVE image at their respective spatial locations, as explained in the material information array preparation section. At the end of this pre-processing step, we get a higher-order tensor ${\mathscr {X}} \in {\mathbb {R}}^{n_s \times n_w \times n_h \times n_m}$ containing $n_m$ layers for each image representing different properties of interest. The input (${\mathscr {X}}_{bw}$), material information arrays (${\mathscr {X}}$) and labels (${\mathscr {Y}}$) of the dataset are shown schematically in Fig. 1.

**Figure 2:**
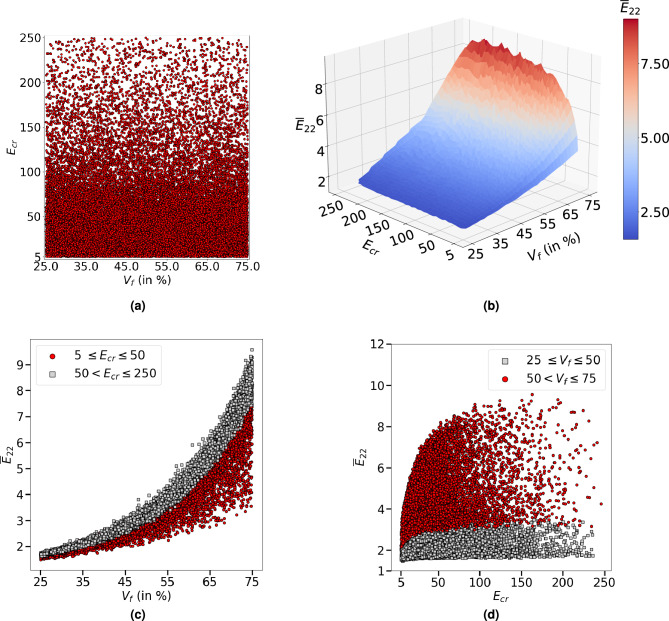
Characteristics of data set ${\mathscr {D}}_1$. (**a**) The distribution of $V_f$ and $E_{cr}$ with 30,000 RVEs, (**b**–**d**) Normalised transverse elastic property ${\overline{E}}_{22} = E_{22}/E_m$ variation with $V_f$ and $E_{cr}$. Note that ${\overline{E}}_{22}$ varies rapidly at lower $E_{cr}$ and higher $V_f$ as indicated by red colour bubbles in (**c**) and (**d**).

In order to develop a generic surrogate model that is applicable to broader practical applications, data sets are created with a wide range of fibre volume fractions $(V_f \in [25\%, 75\%])$ and constituent material property contrasts ($E_{cr} = E_f/E_m \in [5, 250]$). For a given $V_f$, from Adam and Doner^30^ observations, transverse elastic properties of unidirectional composites rapidly increase at lower fibre-matrix elastic modulus contrast $E_{cr}=E_f/E_m$ and then stabilises; this phenomenon becomes more pronounced at higher $V_f$. Transverse elastic modulus is found to stabilise at about $E_{cr}=250$ for $V_f=75\%$^30^ so the maximum $E_{cr}$ selected as 250 in this study. For each RVE, the fibre volume fraction ($V_f$) and material properties ($E_f$ and $E_m$) are drawn randomly with uniform probability from their respective range. If the randomly chosen $E_f$ and $E_m$ are such that $E_{cr}$ is outside the range, a new pair is drawn until the $E_{cr}$ is within its selected range. The scatter plot of $V_f$ and $E_{cr}$ for 30,000 RVEs is shown in Fig. 2a. One can notice that the samples are spread uniformly with respect to fibre volume fraction but non-uniformly with respect to $E_{cr}$. This is due to wider range of $E_{f} \in [10 ~\text {GPa}, 500~\text {GPa}]$ compared to $E_{m} \in [1~\text {GPa}, 10~\text {GPa}]$ with a constraint on the $E_{cr}$ range. For a given $V_f$, from Adam and Doner^30^ and Fig. 2b, transverse elastic property varies rapidly at lower $E_{cr}$ and stabilises at higher $E_{cr}$. Hence, we assume that having lesser samples in the region of negligible property variation has minor effect on the model performance.

The data set, ${\mathscr {D}}_1$, developed in this work contains 30,000 samples with input ${\mathscr {X}}_{bw} \in {\mathbb {N}}^{30,000 \times 256 \times 256 \times 1}$ and labels ${\mathscr {Y}}\in {\mathbb {R}}^{30,000 \times 3}$, which will be split in a 2:1 ratio for training and testing performance of the models, respectively. Here, the size of the RVE binary image (i.e., representing matrix with 0 and fibre with 1) is chosen as $256 \times 256$ following a convergence study, as explained in the next section.

Note that the dataset is designed as a union of 120 chunks wherein each chunk containing 250 samples follows the identical distribution (of $V_f$ and $E_{cr}$) as the whole dataset. This is to ensure the identical distribution for the smaller data sets which will be used in convergence studies for finding the optimum image size and optimum training set size. The following points list the steps involved in data sets preparation while the detailed procedure is given in the later part of this section,

For each RVE,

1. Draw $V_f$ and $E_{cr}$ from the selected range;
2. Generate RVE for the respective fibre volume fraction, $V_f$;
3. Save RVE as a black and white binary image, representing matrix with 0 and fibre with 1;
4. Material information arrays are prepared using Eq. (4), from binary image during the prediction;
5. The transverse elastic properties are determined using physics-based simulations and normalised with their respective matrix modulus.

### Microstructural RVE generation

**Figure 3:**
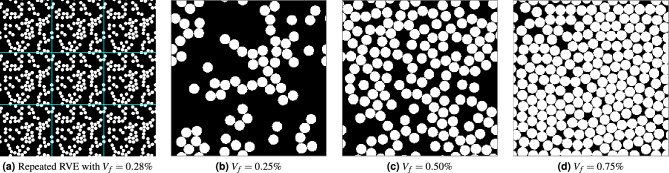
Sample RVE binary images (**a**–**d**), with $256 \times 256$ resolution, at four fibre volume fractions ($V_f$). (**a**) shows the periodicity of the RVEs.

Periodic RVEs of unidirectional composite materials, with the random distribution of circular fibres, are generated using an optimisation-based algorithm recently developed by the authors^31^. Here, the periodicity of RVE implies a fibre leaving an edge(s) must enter from the opposite edge(s) such that RVE is continuous when it is repeated in the space as shown in Fig. 3a. Such periodicity is necessary for applying periodic boundary conditions during the homogenisation of the RVE to evaluate effective properties. RVEs generated using this algorithm have proved randomness in the fibre distribution and transverse isotropy as an actual microstructure using statistical and micro-mechanical analysis^31^. Initially, fibre cross-section centres $\varvec{x} = (x, y)$ are placed randomly in the RVE domain $\Omega$ while allowing fibre overlaps. Then, a constrained optimisation problem is solved to minimise the magnitude of fibre overlap *f* as shown in Eq. (1).

1 $$\begin{aligned} \begin{aligned} \text {Minimize }&f \\ \text {subjected to }\varvec{x}&\in \Omega \end{aligned} \end{aligned}$$

The total magnitude of overlap *f* and its gradient can be explicitly evaluated^31^ as shown in Eq. (2)

2 $$\begin{aligned} \begin{aligned} f&= \sum _{i=1}^{N-1}\sum _{j=i+1}^{N} {C}_{ij}^2 = \sum _{i=1}^{N-1}\sum _{j=i+1}^{N} \left[ ({\overline{d}}_{ij}-d_{ij})\varvec{H}({\overline{d}}_{ij}-d_{ij}) \right] ^2 \\ \dfrac{\partial f}{\partial x_i}&= -2 \sum _{j=1 (\ne i)}^{N} \dfrac{C_{ij}(x_i - x_j)}{d_{ij}} \\ \dfrac{\partial f}{\partial y_i}&= -2 \sum _{j=1 (\ne i)}^{N} \dfrac{C_{ij}(y_i - y_j)}{d_{ij}} \end{aligned} \end{aligned}$$

where $C_{ij}$ is the magnitude of *i*-th fibre intrusion into the *j*-th fibre, $\varvec{H}$ is Heavside step function, $d_{ij}$ is the actual distance of between the centres of fibre *i* and *j*, ${\overline{d}}_{ij}$ is the distance between the centres of fibres when they are externally touching with each other, and *N* is the total number of fibres in the RVE. We have used Julia language^32^ to solve the optimization problem Eq. (1). On a computer with an Intel Xeon CPU 2.40 GHz processor and 64GB RAM, generating 30,000 RVEs with uniform distribution of $V_f \in [25\%, 75\%]$ took 106.8 min. The computational time might vary slightly due to the stochastic nature of $V_f$ and the optimisation convergence for each RVE. Four sample RVE images, generated using this approach, are shown with $256 \times 256$ resolution in Fig. 3.

### Material information array preparation

In this section, the procedure for creating material information arrays from an RVE image is developed. Let the array ${\textbf{I}}^{(g)} \in {\mathbb {R}}^{n_w \times n_h}$ represent a grayscale image of RVE with $N_{ph}$ material phases where a unique pixel value, $p_i \in [0, 1 ] \subset {\mathbb {R}}$, is used to indicate *i*-th phase $\Omega _i$ for $i = 1,2,...,N_{ph}$. In order to avoid confusion with the continuous phase *matrix* of the micro-structure, the term *array* is used to imply a mathematical matrix or, more specifically, rectangular arrangement of image pixel values.

We proceed to construct ${\textbf{I}}^{(\lambda )}$, of same size as ${\textbf{I}}^{(g)}$ but with different pixel values representing material constant or property $\lambda \in [\lambda _{min}, \lambda _{max}]$. The pixel values of ${\textbf{I}}^{(\lambda )}$ can be evaluated using the Eq. (3). Here, the criteria for choosing the bounds, $\lambda _{min}$ and $\lambda _{max}$, need not be based on the admissibility of the property $\lambda$ but rather the range of values used for building the data sets. For example, from Table 1, elastic moduli limits can be chosen as $E_{min}=1$ GPa and $E_{max}=500$ GPa instead of $E>0$ for creating all the data sets.

3 $$\begin{aligned} I_{kl}^{(\lambda )} = \dfrac{1}{\lambda _{max}-\lambda _{min}} \sum _{i=1}^{N_{ph}}\left[ ({\lambda _i - \lambda _{min}}) \delta (I_{kl}^{(g)} - p_i) \right] \end{aligned}$$

where $\delta (x)$ is the Dirac delta function with value 1 for $x=0$ and 0 otherwise. Though Eq. (3) looks involved, it simply normalises the property $\lambda _i$ of *i*-th phase with respect to its bounds to [0, 1].

**Figure 4:**
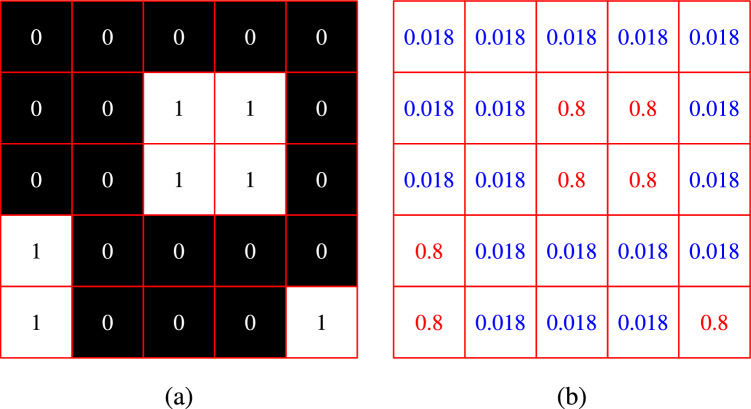
Schematic representation of the material array preparation of two-phase material (**a**) binary image, ${\textbf{I}}^{(g)}$, showing matrix and fibre material, respectively, by 0 and 1 (**b**) elastic modulus array, ${\textbf{I}}^{(E)}$, prepared with $E_{matrix}=10$ GPa, $E_{fibre}=400$ GPa, $E_{min}=1$ GPa and $E_{max}=500$ GPa.

**Figure 5:**
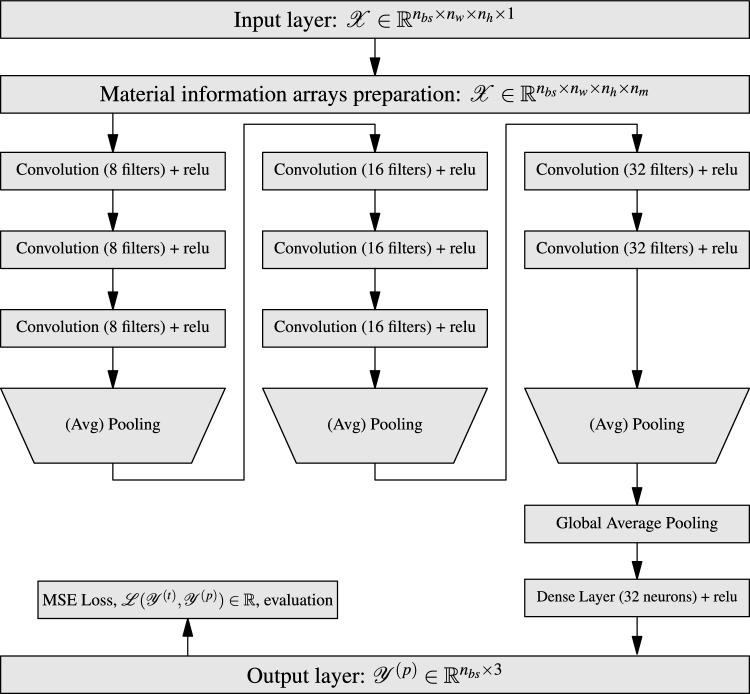
Schematic of the CNN model. Here, $n_{bs}$ is the batch size, and $n_m$ is the number of material information arrays (each having $n_w$ rows and $n_h$ columns), ${\mathscr {Y}}^{(t)}$ and ${\mathscr {Y}}^{(p)}$ are true and predicted values.

In the special case of a two-phase material, the Eq. (3) can be simplified to the Eq. (4). Let the phase $\Omega _1$ and the phase $\Omega _2$ of ${\textbf{I}}^{(g)}$ are represented with pixel values 0 and 1, respectively. Then the whole array, ${\textbf{I}}^{(\lambda )}$, representing the information $\lambda _1$ for phase $\Omega _1$ and $\lambda _2$ for phase $\Omega _2$, can be obtained using the following Eq. (4).

4 $$\begin{aligned} {\textbf{I}}^{(\lambda )} = \dfrac{\lambda _1 - \lambda _{min}}{\lambda _{max} - \lambda _{min}}{\textbf{J}} + \dfrac{\lambda _2 - \lambda _1}{\lambda _{max} - \lambda _{min}}{\textbf{I}}^{(g)} \end{aligned}$$

where ${\textbf{J}} \in {\mathbb {R}}^{n_w \times n_h}$ is an array of all ones. A schematic of the elastic modulus information array, evaluated using Eq. (4), is shown in Fig. 4. It’s worth emphasising that caution must be exercised while saving the material information arrays in image format. Pixel values are generally stored as a byte (8-bit), taking the *integer* values in [0, 255]. This might lead to 256 discrete divisions on the selected range of the material property instead of continuous values, as float values are rounded off to integers. To avoid this trouble, we chose to evaluate material information arrays during the model prediction in the pre-processing stage of the model, as shown in Fig. 5, at the cost of a slight increase in the computational cost.

**Table 1:** Range of fibre volume fraction $V_f$, matrix elastic modulus $E_m$, fibre elastic modulus $E_f$ and elastic moduli contrast ratio $E_{cr}$ of the data sets ${\mathscr {D}}_1$, ${\mathscr {D}}_2$, ${\mathscr {D}}_3$ and ${\mathscr {D}}_4$. $*$ indicates test set MAPE of the respective data set when tested on the model trained with 10,000 samples of ${\mathscr {D}}_1$ without bounds enforcement.

	${\mathscr {D}}_1$	${\mathscr {D}}_2$	${\mathscr {D}}_3$	${\mathscr {D}}_4$
Training set size	20,000	–	–	–
Testing set size	10,000	5000	1500	1500
$V_f$ (in %)	[25, 75]	[25, 75]	[10, 25]	[10, 25]
$E_f$ (in GPa)	[10, 500]	[10, 500]	[10, 500]	[10, 500]
$E_m$ (in GPa)	[1, 10]	[1, 10]	[1, 10]	[1, 10]
$E_{cr}$	[5, 250]	[250, 500]	[250, 500]	[5, 250]
$MAPE^{*}$ (in %)	1.626	2.263	2.787	2.347

In the present work, Poisson’s ratio of fibre and matrix is chosen as the same, $\nu _f$ = $\nu _m$ = 0.25, to reduce the complexity of the analysis. However, this assumption is justified due to the weak dependency of Poisson’s ratio mismatch on the transverse elastic properties^33,34^. Hence, Poisson’s ratio information arrays are not included in the input so each sample contains only the elastic modulus information array.

### Target properties evaluation

Target values of the data sets contain the transverse elastic properties ${\overline{E}}_{22}, {\overline{E}}_{33}$ and ${\overline{G}}_{23}$, normalised with the respective matrix modulus. As the number of RVEs (30,000) is relatively larger, a computationally efficient homogenisation technique based on the variational asymptotic method (VAM)^13^ is used in this work. In this approach, the whole effective elastic matrix ${\overline{D}}$ can be evaluated using a single simulation using the Eq. (5a)^13,35,36^

5a $$\begin{aligned} {\overline{D}}&= \dfrac{1}{\Omega }[D_{pp} - D_{n_a p}^T D_{n_a n_a}^{-T}D_{n_a p}] \end{aligned}$$

5b $$\begin{aligned} D_{pp} = \int _{\Omega }{D d\Omega };\;\;\; D_{n_a p}&=\int _{\Omega }{B^T D d\Omega };\;\;\; D_{n_a n_a}=\int _{\Omega }{B^T D B d\Omega } \end{aligned}$$

where $\Omega$ is the volume of the RVE domain; *D* is the material stiffness matrix of the respective phase with size $p \times p$; *B* is a strain-displacement matrix, and $n_a$ is the number of *total active* degrees of freedom (i.e., excluding the dependent degrees of freedom due to periodic boundary conditions). A homogenisation tool, written in *Julia*^32^ language, is developed to evaluate the effective material matrix ${\overline{D}}$ shown in Eq. (5). Note that VAM-based homogenisation also uses FEA for evaluating the terms in Eq. (5b), thus making it capable of capturing the RVE morphology and ensuring the high-fidelity of the solutions. In contrast to the conventional FEA-based implementations^10,11^, where one needs to solve as many boundary value problems (BVP) and post-processing steps as the number of material matrix columns, VAM-based homogenisation gives ${\overline{D}}$ with a single BVP solution. For example, on a computer with an Intel Xeon CPU 2.40 GHz processor and 64 GB RAM, two-dimensional homogenisation of 20 RVEs using plane strain analysis took about 8.3 min with VAM and about 32.5 min with the conventional FEA approach with the same mesh and loading. This gain in terms of computational time becomes more significant in the case of three-dimensional homogenisation.

Generated RVEs are modelled with a perfect interface between the fibre and the matrix. Then, the periodic mesh needed for applying periodic boundary conditions (PBC) is generated, with plane-strain elements, using an open source software, *gmsh*^37^. Then Eq. (5) is employed to find the transverse effective material matrix ${\overline{D}}$. Mesh convergence study, performed at four combinations of the extremes of $V_f \in [25\%, 75\%]$ and $E_{cr} \in [5, 250]$ ranges, has shown that the convergence of transverse elastic properties at about 50–60 thousand elements. Mesh contains a large proportion of quadrilateral elements and triangular elements in smaller proportion ($<2\%$). Next, the optimum RVE size (the ratio of RVE side length to the fibre radius) is determined as 30 following another transverse elastic property convergence study by varying the RVE size.

## Model development

In this section, a CNN model inspired by the VGG architecture^38^ is designed and trained using the data set ${\mathscr {D}}_1$. The data set is split in a 2:1 ratio for model training and testing, respectively. Initially, a convergence study is performed over pixel sizes 32, 64, 128, 256, 512 to find the optimum RVE image size. Then, CNN models are built and trained at the various training set sizes to understand the influence of data set size on the model performance. It is observed that model performance converges at a certain training set size, beyond which performance gain is insignificant compared to the computational cost. Later, the model’s performance is assessed with respect to fibre volume fraction and elastic moduli contrast. The trained model prediction capability is studied in the extrapolated (or unseen) domain. Finally, the physics-based Hashin–Shtrikman bounds are used to quantify and eliminate the predictions which fall outside these bounds.

### Building and training the CNN model

In Ref.^38^, Simonyan and Zisserman have shown increased efficiency with deeper networks where a small kernel size ($3 \times 3$) coupled with delayed pooling operation is used. The CNN architectures with this idea, known as VGG CNN, have been extensively used in different domains, including some micro-structural applications^18,19,24^. The advantage of using a smaller kernel size with increased depth (or more layers) over a big one is to reduce the number of training parameters and probably enhance learning capability as the non-linear activation function is applied more times through the depth. Also, delayed pooling operation minimises information loss. Hence, in the present work, we have adopted the VGG kind of CNN architecture for building the model as shown in Fig. 5. In all the convolution layers, kernel size and stride are fixed to (3, 3) and (1, 1), while the number of filters is shown in Fig. 5 for each convolution operation. Average pooling is chosen with a size of (2, 2) and a stride of (2, 2), following a comparative study with max pooling operation. Activation functions are essential elements in the deep learning model to infuse non-linearity. So, rectified linear unit (relu) activation is applied after every convolution layer. As the model is built to predict continuous real values, linear activation (or no activation) is used on the output layer. Note that as the data sets are too large to fit into the memory, samples are supplied in batches of size $n_{bs}=64$. Model parameters are updated after every passage of a batch, known as an iteration. An epoch constitutes all such iterations, where the complete training data is sent through the model; to compare across the models, the number of epochs is fixed at 200 in this work. The deviation of the model predictions (${\mathscr {Y}}^{(p)}$) from the ground truth values (${\mathscr {Y}}^{(t)}$) of all the samples in a batch is quantified using the mean squared error (MSE) loss function as shown in Eq. (6).

6 $$\begin{aligned} \text {MSE}:= {\mathscr {L}} = \dfrac{1}{n_{bs}}\sum _{i=1}^{n_{bs}} \sum _{j=1}^{3} (y_{ij}^{(t)} - y_{ij}^{(p)})^2 \end{aligned}$$

where $y_{ij}^{(t)}$ and $y_{ij}^{(p)}$ are true and predicted properties of a sample. Then, the Adam optimisation algorithm^39^ is used, with a learning rate of 0.001, for updating the model weights such that the MSE is minimised. These steps are implemented using PyTorch^40^, an open-source deep-learning library with the Python programming interface, for building and training the CNN model. Training a model with the aforementioned hyper-parameters and ten thousand samples took about 80 min on a machine with 32 GB RAM, 3.7 GHz processor and 8 GB NVIDIA GPU RTX-3050.

### RVE image size selection

**Figure 6:**
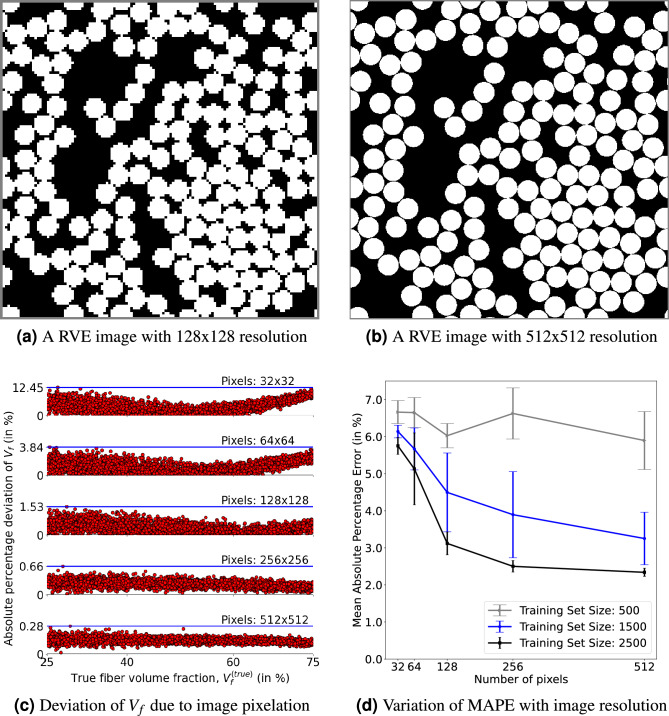
Optimum RVE image size selection. (**a**) and (**b**) shows a sample RVE image with 128 and 512 pixels per side, respectively, wherein the RVE side length is 30 times the fibre radius; (**c**) Absolute percentage deviation of RVE image $V_f$ with true $V_f$ at different resolutions; (**d**) variation of mean absolute percentage error (MAPE) with image resolution.

The computational cost of the model training and inference is directly related to the image size. While a lower image size leads to cheaper computational demand, greedy downsampling of the image might severely alter the micro-structure details. Hence, in this section, we determine the appropriate RVE image size (hence that of material information arrays) by evaluating its influence on the model performance. As the resolution of the image becomes lower, microstructural information may be lost due to pixelation. For example, the RVE of a sample with $54.7\%$ fibre volume fraction is shown in Fig. 6a,b, respectively, with $128 \times 128$ and $512 \times 512$ resolution.

It can be noticed that, with $128 \times 128$, the matrix between two fibre surfaces is replaced with fibre material, and the smooth profile of the fibre cross-section has become coarse. In this study, we consider five different resolutions ($32 \times 32$, $64 \times 64$, $128 \times 128$, $256 \times 256$ and $512 \times 512$) to understand the information loss and its influence on the model training. First, the absolute percentage deviation (APD) of fibre volume fraction due to pixelation of the image is quantified using the Eq. (7) and plotted in Fig. 6c. Here, $V_f^{(image)}$ is evaluated as a fraction of white pixels (representing fibres) in the RVE image. It shows that, for example, saving an RVE at $64 \times 64$ resolution would lead to about 2–4% deviation in $V_f^{(true)}$ if $V_f^{(true)}$ is close to 75%. This deviation is found to reduce by increasing image resolution with less than 1% deviation for resolutions above $256 \times 256$. But, selecting higher resolution causes exponentially increasing computational loads thus higher model training times.

7 $$\begin{aligned} APD = \left| 1 - \dfrac{V_{f}^{(image)}}{V_{f}^{(true)}} \right| \times 100 \end{aligned}$$

Next, models are trained with all five considered resolutions at three different data set sizes (500, 1500, 2500). Further, at each combination of data set size and resolution, ten realisations of models are developed (with the same training samples and hyper-parameters) to account for the statistical nature of the training process. Then, the performance of these models is evaluated on the test samples and quantified with mean absolute percentage error (MAPE); In Fig. 6d, the mean of the MAPE evaluated over ten realisations is plotted against image resolutions with the standard deviation of MAPE as errorbars. It can be noticed that with increasing resolution and training set size, the MAPE and uncertainty have reduced.

From the above analysis, we have selected $256 \times 256$ image resolution for model training as the reduction in $V_f$ deviation (see Fig. 6c) and MAPE (see Fig. 6d) is not significant with an increase in image size from 256 to 512, compared to the increased computational cost.

### Performance evaluation

In order to find the optimum number of samples required for effective learning, different models are trained with the number of samples $n_s \in \{500$, 1000, 1500, 2000, 4000, 6000, 8000, 10,000, 15,000, 20,000$\}$. As explained in the previous section, these subsets of the data set are ensured to have the same kind of distribution as that of the whole data set. Further, to understand the statistical nature of the training process, 10 different realisations of the same model are trained at each of the $n_s$ using the same set of samples and hyper-parameters. So, in total, 100 models are trained with ten subsets of the data set and 10 realisations at each of the subsets. Then, these trained models are tested on samples not seen during the training wherein the test set size is selected as half the size of the training set. In other words, for example, models trained on 5000 samples are tested using 2500 unseen samples. Mean absolute percentage error (MAPE), as defined in Eq. (8), is used to measure the predictive capability of the trained model.

8 $$\begin{aligned} \text {MAPE}_{test, y} = \dfrac{100}{n_{test}} \sum _{i=1}^{n_{test}}{ \left| \dfrac{y_i^{(t)}-y_i^{(p)}}{y_i^{(t)}}\right| } \end{aligned}$$

where $n_{test}$ is the number of test samples, and the superscripts *t* and *p* indicate true and predicted values of *y*. Though MAPE is simpler to interpret and scale independent, it has certain limitations like tending to infinity or undefined when the true value approaches or equals to zero. However, in the present work, normalisation of the effective properties with the respective matrix modulus eliminates such trouble as true or target values $y_i^{(t)}$ are always greater than or equal to one. Also, it is important to note that the absolute percentage error treats underestimation and overestimation differently.

**Figure 7:**
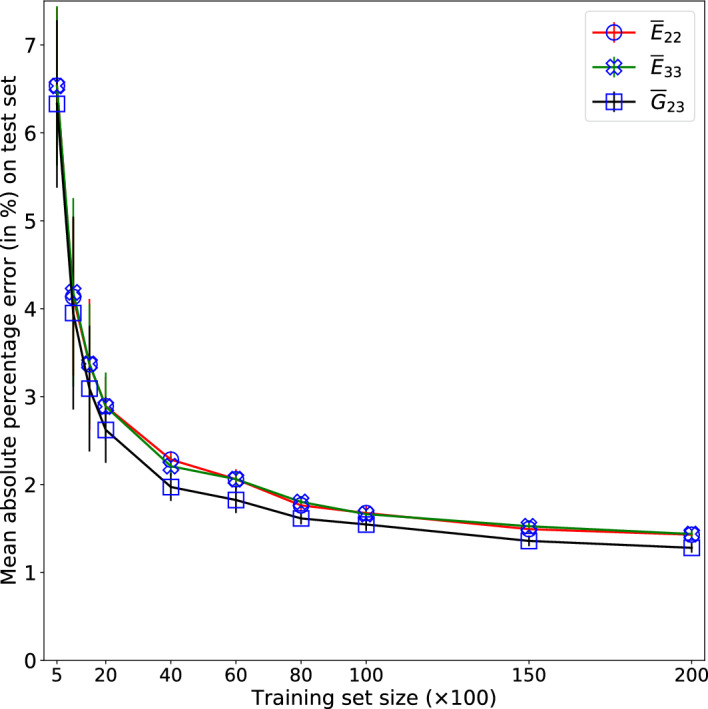
Learning convergence curves of the models trained on the data set ${\mathscr {D}}_1$ showing the variation of each property’s MAPE with training set size. Errorbars indicate the standard deviation of MAPE over ten realisations of the model which are trained with same set of samples and hyper-parameters.

The variation of the mean and standard deviation of MAPE, evaluated on the test set over 10 realisations, is plotted against the number of training examples in Fig. 7. We refer to these curves as learning convergence curves (LCC). In Fig. 7, one can observe that MAPE of all three normalised transverse properties (${\overline{E}}_{22}$, ${\overline{E}}_{33}$, ${\overline{G}}_{23}$) has converged at about a training set of 10,000 samples. Also, as indicated by the error bars, the standard deviation has reduced significantly with the training set size. From this convergence analysis, we have selected training set size of 10000 as optimum and proceed to rigorously analyse the models trained with this data set size.

**Figure 8:**
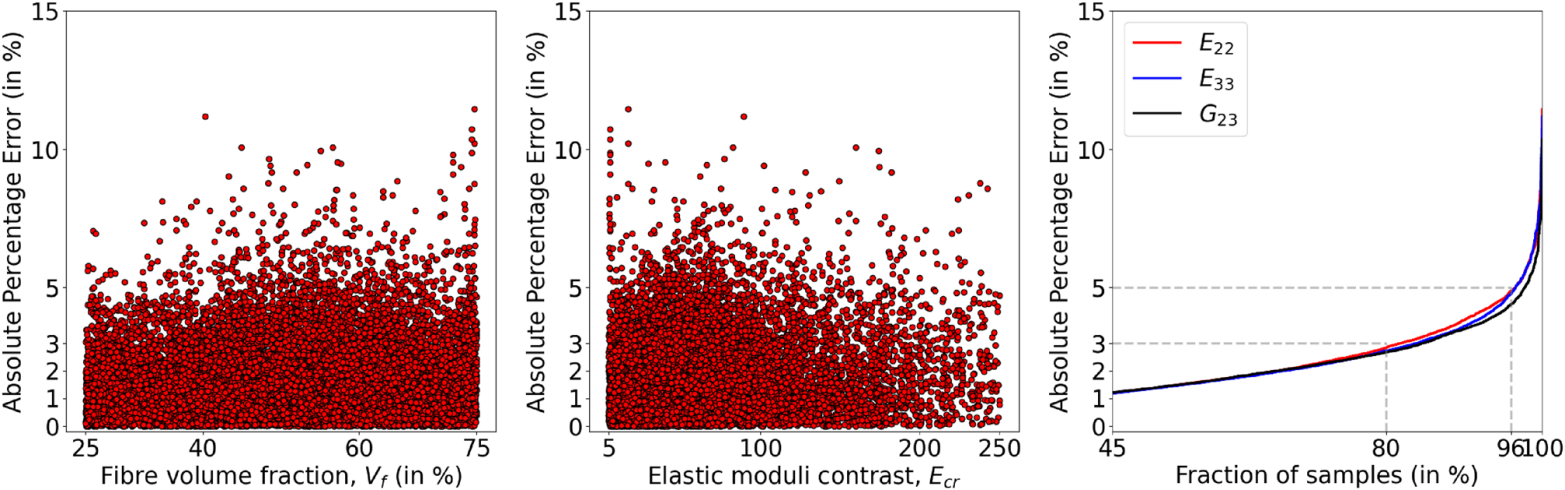
The scatter plots show the absolute percentage error (APE) of target property predictions, on 5000 test samples, with $V_f$ and $E_{cr}$. The cumulative distribution function on the right side shows the fraction of samples under a particular APE; For example, it shows the APE of the model prediction is less than 5% on 97% of the test samples.

The transverse elastic properties (i.e., target properties) depend on the fibre volume fraction $V_f$ and elastic modulus contrast $E_{cr}$, as shown in Fig. 2. It is difficult to infer model performance with respect to these parameters using MAPE, as it squashes information at all $V_f$ or all $E_{cr}$ into a single value, see Eq. (8). So, in order to get a clear understanding of the model’s predictive capability, the absolute percentage error (APE) of each prediction will be studied. In Fig. 8, scatter plots show the APE of all three property predictions for 5000 test samples with respect to $V_f$ and $E_{cr}$. It can be noticed that, except few outliers, the absolute percentage error lies below 5%. The cumulative distribution function on the right side of Fig. 8 shows the fraction of samples below a particular APE. For example, 86% of samples have absolute prediction error less than 3% and below 5% APE for 97% of the test samples.

### Extraterritorial performance

**Figure 9:**
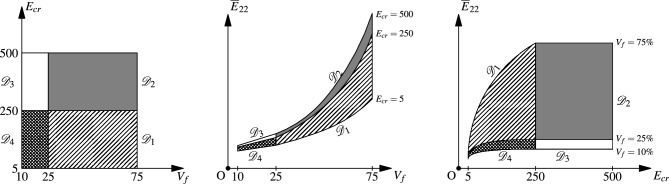
Schematic representation of the three extrapolated domains (with data sets ${\mathscr {D}}_2$, ${\mathscr {D}}_3$ and ${\mathscr {D}}_4$) along with the domain of main data set ${\mathscr {D}}_1$. Note that the fluctuations in ${\overline{E}}_{22}$ at the higher $V_f$ and $E_{cr}$ are not indicated.

**Figure 10:**
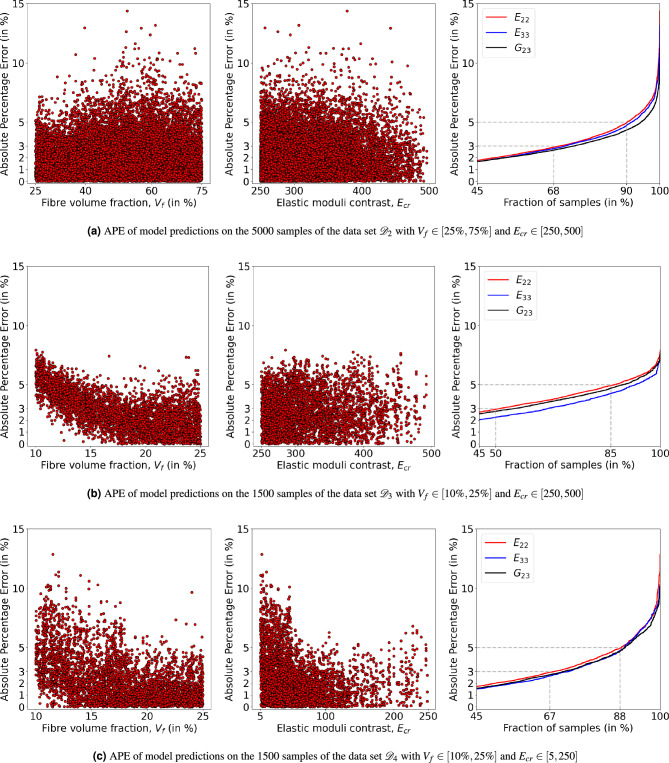
The absolute percentage error (APE) of the model predictions when tested in extrapolated domains ${\mathscr {D}}_2$, ${\mathscr {D}}_3$ and ${\mathscr {D}}_4$. In each of (**a**–**c**) subplots, first two scatter plots show the APE of all three properties with respect to fibre volume fraction $V_f$ and elastic moduli contrast $E_{cr}$. The cumulative distribution function of APE is shown on the right hand side.

In the preceding sections, the surrogate model is built and trained to predict in a wide range of $V_f \in [25\%, 75\%]$ and $E_{cr} = E_f/E_m \ in [5, 250]$. Also, these models are tested on unseen samples which belong to the same range, and the performance is found to be within acceptable levels. It would be interesting to see how the model performs in the extrapolated domain which was not considered during the training. In Fig. 9, extrapolated domains of data sets (${\mathscr {D}}_2$, ${\mathscr {D}}_3$ and ${\mathscr {D}}_4$) with respect to the domain of main data set ${\mathscr {D}}_1$ are shown schematically. In these extrapolated domains, the variation in the property is not significant from its connecting region of the native domain, as shown in the middle and right schematic of Fig. 9. So, the model is expected to predict with reasonably good accuracy as in the native domain. Importantly, such an exercise will help in evaluating the generality of the CNN model and its ability to predict properties of completely unseen microstructures whose characteristics are not present in the training dataset. For testing the model’s performance in these extraterrestrial domains, the size of data sets is selected in proportion to that of the domain size. As the range of $E_{cr}$ is approximately the same for all the domains, the number of test samples is calculated based on the $V_f$ range. For data sets ${\mathscr {D}}_1$ and ${\mathscr {D}}_2$, with 50% $V_f$ range, 5000 test samples are used, and for the remaining two data sets which have 15% $V_f$ range, 1500 test samples are used. The APE of the model predictions on these data sets is shown in Fig. 10, with respect to $V_f$ and $E_{cr}$, along with the cumulative distribution function of APE. In the case of ${\mathscr {D}}_3$ and ${\mathscr {D}}_4$, as shown in Fig. 10b,c, APE shows an increasing trend with decreasing $V_f$. This could be due to deviation in structural information of RVE with decreasing $V_f$, though its target property is not changing significantly. In all three extrapolated domains, the APE of model predictions for at least 85–90% of the test samples is less than 5%. This suggests that the trained model can be used in the extraterritorial domain of $V_f$ and $E_{cr}$.

### Influence of physics-based bounds

**Figure 11:**
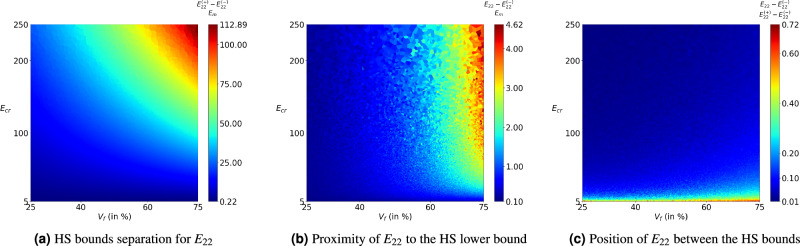
The variation of Hashin–Shtrikman bounds of the data set ${\mathscr {D}}_1$ with fibre volume fraction $V_f$ and elastic moduli contrast $E_{cr}$. (**a**) shows the extent of separation between the bounds normalised with matrix moduli $E_m$; (**b**) and (**c**) shows that the effective property $E_{22}$ lies very close to the lower bound.

In the previous sections, we analysed model performance on the unseen samples of the trained data set domain and on the data sets of extrapolated domains. It is observed that the absolute percentage error of the predictions is within the acceptable limits. However, model predictions may or may not be physically admissible. In this section, the admissibility of these predictions is assessed using the physics-based bounds available in the literature^29^. We use simpler and relatively tighter Hashin–Shtrikman (HS) bounds^28^, which can be evaluated using the Eq. (10). In general, the lower and upper bounds on the effective properties of the composite material are separated by a large magnitude, as shown in Fig. 11a. It can be noticed that the bounds get wider with increasing $V_f$ and contrast ratio $E_{cr}$. And, the transverse properties lie closer to the lower bound ( as shown in Fig. 11b,c), thus there is a possibility that the model prediction might go out of the lower bound.

9 $$\begin{aligned} K^{(-)}&= K_m + \dfrac{V_f}{\dfrac{1}{K_f - K_m} + \dfrac{1-V_f}{K_m + G_m}}\quad \quad G^{(-)} = G_m + \dfrac{V_f}{\dfrac{1}{G_f - G_m} + \dfrac{(1-V_f)(K_m + 2G_m)}{2G_m(K_m + G_m)}}\quad \quad E^{(-)} \nonumber \\&= \dfrac{9 K^{(-)}G^{(-)}}{3K^{(-)} + G^{(-)}} \end{aligned}$$

10 $$\begin{aligned} K^{(+)}&= K_f - \dfrac{1-V_f}{\dfrac{1}{K_f - K_m} - \dfrac{V_f}{K_f + G_f}} \quad \quad G^{(+)} = G_f - \dfrac{1-V_f}{\dfrac{1}{G_f - G_m} - \dfrac{V_f(K_f + 2G_f)}{2G_f(K_f + G_f)}} \quad \quad E^{(+)} \nonumber \\&= \dfrac{9 K^{(+)}G^{(+)}}{3K^{(+)} + G^{(+)}} \end{aligned}$$

where suffix *f* and *m* refer to fibre and matrix, *K* is the bulk modulus, *G* is shear modulus, *E* is Young’s modulus, super-fix $(-)$ and $(+)$ indicate lower and upper bounds.

The number of outliers to HS lower bounds is evaluated on all 10 realisations of the model, which are trained on 10,000 samples of the data set ${\mathscr {D}}_1$. The maximum number of outliers for each property with all four data sets is listed in Table 2.

**Table 2:** On the model trained *without* bounds enforcement, number of predictions which lie below the lower bound of the respective property, when tested on unseen samples of the four data sets ${\mathscr {D}}_1$, ${\mathscr {D}}_2$, ${\mathscr {D}}_3$, ${\mathscr {D}}_4$. Numbers here indicate the maximum number of outliers over ten realisations of the models. On the model trained *with* bounds enforcement, the number of outliers are reduced to zero.

Property	Number of outliers to the data sets			
	${\mathscr {D}}_1$	${\mathscr {D}}_2$	${\mathscr {D}}_3$	${\mathscr {D}}_4$
$E_{22}$	0	0	533	651
$E_{33}$	0	0	251	318
$G_{23}$	5	0	862	796

**Figure 12:**
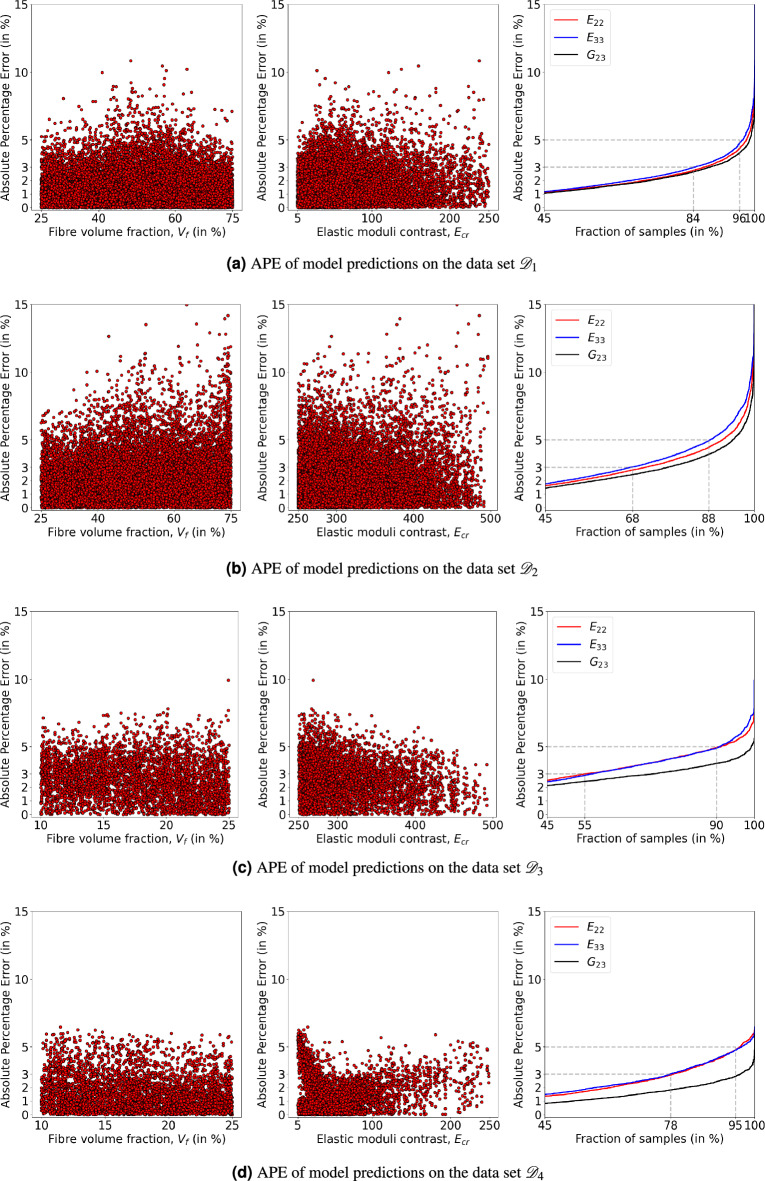
Absolute percentage error (APE) of the bounds-enforced model predictions when tested on the data sets ${\mathscr {D}}_1$, ${\mathscr {D}}_2$, ${\mathscr {D}}_3$ and ${\mathscr {D}}_4$. In (**a**–**d**), first two scatter plots indicate the APE of model predictions with respect to fibre volume fraction $V_f$ and elastic moduli contrast $E_{cr}$. On the right-hand side, the cumulative distribution function of APE shows the fraction of samples below 3% APE and 5% APE.

It shows that a large number of model predictions on the data sets ${\mathscr {D}}_3$ and ${\mathscr {D}}_4$ are below the lower bound. Now we proceed to enforce these bounds during the model training such that all the model predictions fall within the bounds. While training a model, in general, bounds can be enforced in two ways. In the first approach, known as soft enforcement, the loss function of the model is regularised by weighed addition of the mean square errors of the deviation of the predictions from the bounds. Generally, the weights of these additional loss terms are hyper-parameters which need to be tuned manually. In the second approach, known as hard enforcement, the model predictions are transformed to lie within the bounds thereby avoiding additional hyper-parameters. In the present work, we chose to enforce bounds in a hard manner. In this approach, model architecture and training are similar to the one shown in Fig. 5, except few changes at the end of the network. The output of the network’s last layer is mapped to $[-1, 1]$ by applying the $\tanh$ activation function. Then, these values are further scaled to lie between lower and upper bounds as shown in Eq. (11). It’s worth mentioning that the model outputs are *not* constrained to bounds, but the model is trained to predict values between the bounds.

11 $$\begin{aligned} y^{(p)} = y^{(-)} + \dfrac{1 + y^{*}}{2}\left[ y^{(+)} - y^{(-)}\right] \end{aligned}$$

where $y^{*} \in [-1, 1]$ is the output of $\tanh$ activation function on the last layer, $y^{(-)}$ and $y^{(+)}$ are lower and upper bounds. It is observed that, unlike without bounds training, the training with bounds is sensitive to the learning rate; bounds-enforced models are trained with an optimal learning rate of 0.0005. The overall MAPE of the model predictions, after 200 epochs, is about 1.72 in the same range as with models trained without bounds (see Table 1). Nevertheless, the absolute percentage error of the predictions in the extrapolated domains ${\mathscr {D}}_3$ and ${\mathscr {D}}_4$ is improved, as shown in Fig. 12, in addition to eliminating the number of outliers, for all the domains. It suggests that, for predictions in the extrapolated domain, especially towards the lower fibre volume fractions, enforcing the bounds is important in predicting physically valid properties.

## Conclusions

CNN models are developed for predicting the normalised transverse elastic properties of fibre-reinforced composites. In order to increase the applicability of the model, it is trained on a wide range of fibre volume fractions in [25%, 75%] and fibre-matrix elastic modulus contrast ratio in [5, 250]. The model is shown to provide very good predictions even on completely unseen microstructures that lie outside of the considered range of volume fractions (in [10%, 25%]) and modulus ratios (in [250, 500]). Further, the study demonstrated that careful data set preparation and training design is crucial for achieving better model performance. In summary,

- A simple and novel method is developed for encoding material properties of constituents in the greyscale image of the micro-structure so that the model learns material information along with the geometric information.
- RVE binary image with a resolution of $256 \times 256$ is found to have minimum $V_f$ deviation ($<1\%$) from true $V_f$; Also, MAPE is found to have converged at this RVE image resolution.
- Stochastic nature of the training process is quantified using the mean and standard deviation of MAPE, evaluated on 10 realisations of the model training.
- Using the learning convergence curves, the optimum training set size is determined as ten thousand beyond which reduction in MAPE of model predictions is found to be negligible.
- In the training set domain, at least 96% of the 5000 test sample predictions have absolute percentage error (APE) less than 5%.
- In the case of extrapolated domains, at least about 85–90% of the test samples have APE less than 5%.
- At the end, we have trained the models with hard enforcement of the physics-based HS bounds such that the model predictions are always physically admissible. Also, this has improved the model’s performance metric APE in the extrapolated domains ${\mathscr {D}}_3$ and ${\mathscr {D}}_4$.

The proposed material encoding idea can be employed to build surrogate models for heterogeneous, anisotropic materials of varied constituent combinations by using the stack of relevant material information arrays as input to the network. Also, as the model spans a wide range of fibre volume fractions and elastic modulus contrasts, the trained models can be used in the inverse design of the microstructures which gives the properties of interest.

## Data Availability

The datasets used and/or analysed during the current study are available at the following link https://github.com/338rajesh/mpi-cnn.
